# Prévalence et caractéristiques des effets indésirables des antihypertenseurs chez les patients suivis en ambulatoire au Centre Hospitalier Universitaire Yalgado Ouédraogo

**DOI:** 10.11604/pamj.2018.29.84.13754

**Published:** 2018-01-29

**Authors:** Georges Rosario Christian Millogo, Ragomzingba Frank Edgard Zongo, Anita Benao, Estelle Noëla Hoho Youl, Blaise Alexandre Bazona Bassoleth, Moussa Ouédraogo, Patrice Zabsonré, Innocent Pierre Guissou

**Affiliations:** 1 Unité de Formation et de Recherche en Sciences de la Santé, Université Ouaga I Professeur Joseph Ki Zerbo, Burkina Faso; 2 Département de Médecine, Service de Cardiologie, Centre Hospitalier Universitaire Yalgado Ouédraogo, Burkina Faso; 3 Département Pharmacie Hospitalière, Centre Hospitalier Universitaire Yalgado Ouédraogo Burkina Faso

**Keywords:** Hypertension artérielle, antihypertenseur, effet indésirable, CHUYO, Burkina Faso, Arterial hypertension, antihypertensive, adverse effect, University Hospital Yalgado Ouédraogo (CHUYO), Burkina Faso

## Abstract

La prise en charge médicamenteuse de l'hypertension artérielle (HTA) entraine des effets indésirables qui peuvent être gênants et ainsi influencer l'observance du patient. Nous avons étudié ces effets indésirables dans le service de cardiologie du Centre hospitalier universitaire Yalgado Ouédraogo afin de déterminer leurs fréquences et leurs caractéristiques. Il s'agissait d'une étude transversale de juillet à septembre 2015 chez les patients suivis en ambulatoire pour HTA. Les données ont été obtenues à partir de l'interrogatoire, des carnets de suivi des patients et des fiches de consultations. Au total 278 patients ont été inclus. La population d'étude incluait 69,1% de femmes. L'âge moyen était de 52,2 ans avec des extrêmes de 23 et 86 ans. Quatre vingt et sept virgule huit pourcent (87,8%) vivaient en milieu urbain. Le tabagisme, la dyslipidémie et les antécédents familiaux d'HTA représentaient respectivement 9%, 35,6% et 57,2%. Au plan thérapeutique, 43,2% étaient sous monothérapie, 35,6% sous bithérapie à l'initiation du traitement. Les inhibiteurs calciques (59,7%) étaient la classe thérapeutique la plus utilisée. La prévalence globale des effets indésirables était de 60,1%. Les inhibiteurs calciques étaient impliqués dans 53,6% suivis des diurétiques (48,6%) dans la survenue de l'effet indésirable. La prévalence spécifique par molécule était 28,1% pour l'amlodipine et 24,5% pour l'hydrochlorothiazide. La diurèse excessive (13,7%), la toux (12,9%) et les vertiges (11,5%) étaient les effets indésirables les plus fréquemment évoqués par les patients. Le système nerveux central et périphérique et le système ostéo-musculaire étaient les systèmes les plus atteints. Les effets indésirables sont un déterminant majeur de l'adhésion aux traitements antihypertenseur, car leur impact sur la vie quotidienne des patients peut s'avérer significatif.

## Abstract (en)

Medicinal treatment of arterial hypertension (AH) may cause adverse effects which can be annoying and thus influence patient’s compliance with treatment. Our study focused on these undesirable effects in patients followed up for AH on an ambulatory basis in the Department of Cardiology at the University Hospital Yalgado Ouédraogo from July to September 2015. Our aim was to determine their rates and their characteristics. Data were obtained from patients’ interview, from the examination of patients’ follow-up records and of medical consultation forms. A total of 278 patients were included in the study, 69.1% of them were women. The average age was 52.2 ranging between 23 and 86 years; 87.8% of patients lived in urban areas. Smoking, dyslipidemia and a family history of AH accounted for 9%, 35.6% and 57.2% of cases respectively. From a therapeutic perspective, 43.2% of patients were under monotherapy, 35.6% under bitherapy at the start of treatment. Calcium channel blockers (59.7%) were the most used therapeutic drugs. The overall incidence of adverse effects was 60.1%. Calcium channel blockers were involved in 53.6% of adverse events of patients, diuretics in 48.6%. Molecule-specific prevalence was 28.1% for the amlodipine and 24.5% for the hydrochlorothiazide. Excessive diuresis (13.7%), cough (12.9%) and vertigo (11.5%) were the most frequent adverse events reported by the patients. The central and peripheral nervous system and the osteo-muscular system were the most affected systems. Adverse effects are a major determinant of patient’s compliance with antihypertensive treatments, because they may have a significant impact on patient’s daily life.

## Introduction

Les maladies chroniques non transmissibles dont les maladies cardio-vasculaires sont en pleine progression dans le monde. L'hypertension artérielle (HTA), facteur majeur de risque de morbidité et de mortalité cardiovasculaire, constitue un véritable problème de santé publique mondial [1] et en Afrique Noire [2-4]. Au Burkina Faso, les chiffres de prévalence en milieu urbain étaient estimés à 23% en 2003 [5], 30% en 2010 [6] et 29,6% en 2015 [7]. La prise en charge de l'HTA, selon les recommandations de l'OMS, met l'accent sur les mesures hygiéno-diététiques [8-10]. Toutefois, l'option d'un traitement médicamenteux s'impose dans de nombreux cas. Aujourd'hui, plusieurs classes pharmacologiques sont disponibles pour le traitement de l'HTA. Cependant, ces traitements médicamenteux entrainent de nombreux effets indésirables qui rendent souvent l'adhésion au traitement très difficile. En effet, avant la mise sur le marché des médicaments, la connaissance des effets indésirables n'est pas exhaustive. Au cours des essais cliniques, le médicament n'a été administré qu'à un nombre limité de sujets (quelques centaines à quelques milliers) sélectionnés et soumis à une surveillance soutenue. Dans ces conditions, les effets indésirables rares et potentiellement graves ont statistiquement peu de chance d'être observés [11]. Une bonne connaissance de ces effets indésirables permet d'une part, de faire le meilleur choix dans l'option thérapeutique et d'autre part, d'éduquer le patient sur la survenue des effets indésirables ainsi que sur leur gestion afin d'améliorer l'adhésion et l'observance du traitement par le patient. Ainsi, en vue de contribuer à l'amélioration de la prise en charge de l'HTA dans notre contexte, nous avons entrepris d'étudier les effets indésirables des antihypertenseurs chez les patients hypertendus suivis en ambulatoire dans le service de cardiologie du centre hospitalier universitaire Yalgado Ouédraogo (CHUYO). Cette étude vise à déterminer la prévalence des effets indésirables des médicaments antihypertenseurs chez les patients hypertendus suivis en ambulatoire dans le service de cardiologie du CHUYO d'une part, et d'autre part décrire les caractéristiques de ces effets indésirables.

## Méthodes

**Population:** Nous avons réalisé une enquête transversale auprès des patients adultes reçus dans le service de cardiologie, l'un des services du département de médecine et spécialités médicales du CHUYO, le plus grand hôpital universitaire de référence du Burkina Faso. L'enquête a été réalisée sur une période de 3 mois allant du 1^er^ Juillet 2015 au 30 Septembre 2015.

### Sélection des patients

**Critères:** Nous avons inclus dans l'enquête, les patients âgés de plus de 15 ans suivis en ambulatoire pour une HTA non compliquée et traités par au moins un antihypertenseur du 1^er^ Janvier 2015 au 30 Juin 2017. Le consentement écrit du patient était exigé avant le début de l'interview. Les nouveaux patients ou les patients consultant pour la première fois n'ont pas été inclus dans l'étude.

**Echantillonnage:** Un échantillonnage aléatoire a été fait sur la base d'une liste de patients ayant consulté dans l'année précédant le début de l'enquête. Cette liste a été établie à partir du registre de consultations des patients ambulatoires. A partir de cette liste de patients (et de leur numéro de téléphone) classée par ordre alphabétique, un tirage aléatoire simple a été réalisé. Avec une prévalence attendue de 30% d'effets indésirables nous avons estimé une taille d'échantillon de 81 patients à enquêter (α = 5%, précision 10%).

**Variables:** Les variables de l'étude étaient: les caractéristiques du patient: âge, sexe, profession, antécédents; Les données cliniques et thérapeutiques: poids, taille, grades selon l'organisation mondiale de la santé (OMS) de l'Indice de masse corporelle (IMC) - normal -' surpoids - obésité classes 1, 2 et 3 -, tabagisme, alcoolisme, date de découverte de l'HTA, circonstance de découverte de l'HTA, nombre de molécules composant le traitement, changement de médicament, motif du changement du traitement, autres médicaments pris par le patient); Les évènements indésirables l'événement indésirable est défini comme étant «toute manifestation nocive et non recherchée survenant chez une personne pendant ou après un traitement, qu'elle soit considérée ou non comme liée à un ou des produit(s) de santé à usage humain »; Les effets indésirables: nature, fréquence, prévisibilité (Effet indésirable inattendu: effet indésirable non décrit dans le résumé des caractéristiques du produit (RCP) ou la notice du médicament - Effet indésirable attendu: effet indésirable décrit dans le RCP ou la notice du médicament), gravité (grave, sévère, modéré/banal), évolution (guérison, guérison avec séquelles, décès, pas encore guéri, inconnue); L'effet indésirable grave est définit comme un effet indésirable létal, ou susceptible de mettre la vie en danger, ou prolongeant une hospitalisation, ou se manifestant par une anomalie ou une malformation entrainant une invalidité ou une incapacité importante ou durable, ou provoquant ou prolongeant une hospitalisation, ou se manifestant par une anomalie ou une malformation congénitale.

**Collecte et traitement des données:** Les patients tirés ont été contactés par téléphone. Le but et les objectifs de l'étude leur ont été expliqués. Puis, un rendez-vous était pris avec le patient pour l'administration du questionnaire après recueil du consentement. Les données ont été collectées à l'aide d'un questionnaire administré à chaque patient et en exploitant les renseignements disponibles dans le dossier médical du patient, le carnet de suivi du patient et les résultats des examens biologiques détenus par le patient. En cas de signalement par le patient d'un évènement indésirable survenu au cours du traitement antihypertenseur, la fiche de pharmacovigilance était remplie. Puis, l'imputabilité selon la méthode OMS était réalisée. L'événement indésirable pour lequel le score d'imputabilité pour un médicament du traitement antihypertenseur était égal à « possible », « probable » ou « certain » était considéré comme un effet indésirable. La définition de l'effet indésirable est celle de l'OMS.

**Analyses statistiques:** Nous avons utilisé le logiciel Epi Info 7 en plus du logiciel Excel version 6. Les résultats sont présentés en effectif et en pourcentage pour les variables qualitatives, et en moyenne et écart type pour les variables quantitatives.

**Considérations éthiques et déontologiques:** Le consentement éclairé du patient a été demandé et la confidentialité des patients a été respectée tout au long du processus de recueil des données. L'anonymat des patients a été respecté lors du remplissage des fiches de collecte. Nous avons par ailleurs obtenu l'autorisation.

## Résultats

**Caractéristiques sociodémographiques, médicales et thérapeutiques de la population:** Au total 278 patients ont été enquêtés. Le Tableau 1 décrit les caractéristiques sociodémographiques, les facteurs de risques et antécédents pathologiques des patients de l'étude. Respectivement 176 et 88 patients ont fait l'objet d'un changement de traitement antihypertenseur et deux changements de traitement. Le Tableau 2 indique la répartition des patients selon les molécules utilisées lors du traitement initial puis du premier et du deuxième changement du traitement antihypertenseur.

**Tableau 1:** Caractéristiques des patients enquêtés (n=278)

Caractéristiques	Fréquence	
	n	%
**Caractéristiques sociodémographiques**		
**Sexe**		
Féminin	192	69,1
Masculin	86	30,9
**Age (années) (moyenne 52,1)**		
˂ 30	7	2,5
[30-40[	34	12,2
[40-50[	58	20,9
[50-60[	94	33,8
[60-70[	62	22,3
[70-80[	20	7,2
≥ 80	3	1,1
**Profession/statut**		-
Fonctionnaire de l’Etat	79	28,4
Femme au foyer	67	24,1
Elève/Etudiant	1	0,4
Secteur informel	63	22,7
Sans emploi	3	1,1
Retraité	53	19,1
Autre	12	4,3
**Résidence**		-
Milieu rural	8	2,9
Milieu semi-urbain	26	9,4
Milieu urbain	244	87,8
**Facteurs de risque et antécédents pathologiques**		-
Tabagisme	25	9,0
Consommation d'alcool	99	35,6
Dyslipidémie	99	35,6
Diabète	28	10,1
**Indice de masse corporelle (IMC)**		-
Obésité classe 1	58	20,9
Obésité classe 2	17	6,1
Obésité classe 3	17	6,1
Normal	108	38,8
Surpoids	78	28,1
Antécédents familiaux d'HTA	159	57,2

**Tableau 2:** Répartition des patients selon les molécules utilisées lors du traitement initial puis du premier et du deuxième changement du traitement antihypertenseur

Caractéristiques du traitement	Traitement initial(n=278)		Premier changement (n=176)		Deuxième changement(n=88)	
	n	%	n	%	n	%
**Nombre de molécules composant le traitement**						
1	120	**43,2**	51	29,0	**17**	19,3
2	99	35,6	72	**40,9**	**35**	**39,8**
3	48	17,3	41	23,3	**24**	27,3
4	11	4,0	12	6,8	**12**	13,6
**Classes et molécules utilisées dans les traitements**						
**Inhibiteurs calciques (comprimé)**	**166**	**59,7**	**93**	**33,5**	**46**	**16,5**
Nifédipine	82	**29,5**	27	9,7	12	4,3
Amlodipine	74	**26,6**	62	**22,3**	29	10,4
Nicardipine	7	2,5	1	0,4	3	1,1
Vérapamil	2	0,7	3	1,1	2	0,7
Lercanidipine	1	0,4	0	-	0	-
**Diurétiques (comprimé)**	**122**	**43,9**	**92**	**33,1**	**62**	**22,3**
Hydrochlorothiazide	63	22,7	47	16,9	30	**10,8**
Furosémide	23	8,3	8	2,9	3	1,1
Spironolactone	13	4,7	25	9,0	16	5,8
Indapamide	12	4,3	4	1,4	5	1,8
Amiloride	6	2,2	4	1,4	5	1,8
Triamtérène	5	1,8	4	1,4	3	1,1
**Inhibiteurs de l'enzyme de conversion (comprimé)**	**110**	**39,6**	**102**	**36,7**	**48**	**17,3**
Captopril	66	23,7	49	17,6	14	5,0
Enalapril	19	6,8	34	12,2	21	**7,6**
Périndopril	15	5,4	11	4,0	9	3,2
Ramipril	10	3,6	7	2,5	2	0,7
Lisinopril	0	0,0	1	0,4	2	0,7
**Bêta-bloquants (comprimé)**	**56**	**20,1**	**50**	**18,0**	**27**	**9,7**
Aténolol	48	17,3	42	15,1	23	**8,3**
Propranolol	3	1,1	3	1,1	0	-
Acébutolol	3	1,1	3	1,1	2	0,7
Bisoprolol	2	0,7	2	0,7	2	0,7
**Antihypertenseurs centraux (comprimé)**	**38**	**13,7**	**13**	**4,7**	**10**	**3,6**
Alpha méthyl dopa	38	13,7	13	4,7	10	3,6
**Anti angiotensine II (comprimé)**	**14**	**5,0**	**16**	**5,8**	**14**	**5,0**
Losartan	10	3,6	14	5,0	14	5,0
Olmesartan	2	0,7	2	0,7	0	-
Ibesartan	2	0,7	0	-	0	-

**Prévalence et caractéristiques des effets indésirables des médicaments antihypertenseurs:** Au total, les effets indésirables ont été observés chez 167 patients soit une prévalence globale de 60,1%. La majorité des patients (58,1%) ont signalé l'effet indésirable lors de l'interview; les effets étaient mentionnés dans le carnet de santé chez 27,5% des patients. Chez 14,4% des patients, ils étaient aussi bien déclarés par les patients et mentionnés dans le carnet de santé par le médecin traitant. Le Tableau 3 donne la répartition des effets indésirables selon les classes pharmacologiques de médicaments et le médicament. Il donne également la prévalence des personnes ayant manifesté un effet indésirable en fonction du système ou appareil atteint. Les Tableau 4, Tableau 4 (suite) et Tableau 4 (suite 1) montrent une répartition des effets indésirables selon le système physiologique affecté.

**Tableau 3:** Caractéristiques des effets indésirables observés chez les patients enquêtés en fonction des molécules anti-hypertensives (n=278)

Caractéristiques	Fréquence	Pourcentage
**Prévalence de l'effet indésirable en fonction de la molécule**		
**Inhibiteurs calciques (comprimé)**	**149**	**53,6**
Amlodipine	78	**28,1**
Nifédipine LP	61	21,9
Nicardipine LP	7	2,5
Vérapamil LP	2	0,7
Lercanidipine	1	0,4
**Diurétiques (comprimé)**	**135**	**48,6**
Hydrochlorothiazide	68	**24,5**
Spironolactone	26	9,4
Furosémide LP	18	6,5
Triamtérène	9	3,2
Amiloride	8	2,9
Indapamide	6	2,2
**Inibiteurs de l'enzyme de conversion (comprimé)**	**134**	**48,2**
Captopril	62	**22,3**
Enalapril	44	15,8
Périndopril	19	6,8
Ramipril	8	2,9
Lisinopril	1	0,4
**Bêta-bloquants (comprimé)**	**66**	**23,7**
Aténolol	54	19,4
Acébutolol	5	1,8
Propranolol	4	1,4
Bisoprolol	3	1,1
**Antihypertenseurs centraux (comprimé)**	**33**	**11,9**
Alpha méthyl dopa	33	11,9
**Anti angiotensine II (**comprimé)	**28**	**10,1**
Losartan	23	8,3
Olmésartan	3	1,1
Ibésartan	2	0,7

**Tableau 4:** Fréquence des effets indésirables en fonction de l’appareil ou système atteint et fréquence des patients ayant manifesté un effet indésirable

Appareil ou système d'atteint	Type d'effet indésirable (EI) manifesté	Fréquence d’EI		Nombre de patients ayant manifesté l’EI (n=167)	Fréquence (%) des patients ayant manifesté l’EI
**Appareil cochléaire et vestibulaire**	Vertiges	**32**	40	38	13,7
	Bourdonnement des oreilles	8			
**Appareil génital féminin**	Diminution de la libido	5	9	9	3,2
	Augmentation du volume des seins	3			
	Galactorrhée	1			
**Appareil génital masculin**	Dysfonction érectile	15	20	19	6,8
	Impuissance sexuelle	3			
	Gynécomastie	2			
**Appareil respiratoire**	Toux sèche	**36**	52	47	16,9
	Dyspnée	12			
	Bronchospasme	1			
	Irritation laryngée	1			
	Rhume	1			
	Sensation de nez bouché	1			
**Appareil urinaire**	Diurèse excessive	**38**	38	38	13,7
**Appareil visuel**	Trouble visuel	8	9	9	3,2
	Douleur oculaire	1			
**Etat général**	Asthénie	12	17	15	5,4
	Malaise	3			
	Douleur diffuse	1			
	Perte de poids	1			
**Fréquence et rythmes cardiaques**	Palpitation	14	38	36	12,9
	Bradycardie	11			
	Hypotension orthostatique	6			
	Tachycardie	6			
	Arythmie d'allure extrasystolique	1			

**Tableau 4(suite):** Fréquence des effets indésirables en fonction de l’appareil ou système atteint et fréquence des patients ayant manifesté un effet indésirable

Appareil ou système d'atteint	Type d'effet indésirable (EI) manifesté	Fréquence d’EI		Nombre de patients ayant manifesté l’EI (n=167)	Fréquence (%) des patients ayant manifesté l’EI
**Métabolisme et nutrition**	Hyperkaliémie	6	19	12	4,3
	Hyperglycémie	2			
	Hypocalcémie	2			
	Hyperaldostérinémie	1			
	Hyperprotidémie	1			
	Hyperuricémie	1			
	Hypoglycémie	1			
	Hypomagnésémie	1			
	Hyponatrémie	1			
	Petite acidose	1			
	Syndrome métabolique	1			
	Trouble électrolytique	1			
**Peau et annexes**	Prurit	11	28	21	7,6
	Sécheresse de la bouche	7			
	Eruptions cutanées	4			
	Perte des cheveux	2			
	Réaction allergique	2			
	Lésions cutanées	1			
	Picotement généralisé	1			
**Système gatro-intestinal**	Douleur épigastrique	7	22	21	7,6
	Nausée	6			
	Mal de gorge	3			
	Constipation	2			
	Douleur gingivale	1			
	Régurgitation	1			
	Trouble du goût	1			
	Vomissement	1			

**Tableau 4(suite 1):** Fréquence des effets indésirables en fonction de l’appareil ou système atteint et fréquence des patients ayant manifesté un effet indésirable

Appareil ou système d'atteint	Type d'effet indésirable (EI) manifesté	Fréquence d’EI		Nombre de patients ayant manifesté l’EI (n=167)	Fréquence (%) des patients ayant manifesté l’EI
**Système nerveux central et périphérique**	Céphalée	19	64	53	19,1
	Paresthésie	17			
	Cauchemar	7			
	Insomnie	6			
	Somnolence	5			
	Sensation de tête vide	4			
	Etourdissement	3			
	Sensation de perte de mémoire	2			
	Trouble du sommeil	1			
**Système ostéo-musculaire**	Crampes	35	59	53	19,1
	Douleur articulaire et musculaire	10			
	Lombalgie	8			
	Douleur thoracique	3			
	Tremblement	3			
**Système vasculaire extra-cardiaques**	Œdèmes au visage et au cou	13	28	27	9,7
	Jambes lourdes et enflées	11			
	Boussiffure du visage	3			
	Œdème cérébral	1			
**Systèmes endocriniens**	Bouffées de chaleur	4	9	8	2,9
	Transpiration excessive	3			
	Déshydratation	2			
**Troubles psychiatriques**	Humeur dépressive	1	2	2	0,7
	Nervosité	1			
**Lignée sanguine rouge**	Anémie hémolytique	2	3	3	1,1
	Anémie normocytaire normochrome	1			
**Lignée sanguine blanche**	Hyperleucocytose	1	1	1	0,4
**Foie et voies biliaires**	Elévation des transaminases-ASAT	1	1	1	0,4
**Autres organes de sens**	Trouble de l'audition	1	1	1	0,4

## Discussion

**Limites de l'étude:** Notre étude comporte des limites pouvant avoir affecté nos résultats. Tout d'abord, le type d'étude, transversale, a pu introduire plusieurs biais au nombre desquels nous pouvons citer: le biais d'information: le fait de se sentir questionné/observé a pu avoir augmenté la déclaration des effets indésirables; Le biais de confusion concernant l'état de santé est aussi possible. Il se pourrait que l'effet indésirable fusse davantage imputable à l'état de santé du patient qu'au médicament consommé; Le biais de mémoire: les patients pourraient ne plus se souvenir ni des effets indésirables survenus lorsqu'il y a eu des changements de molécule, ni de la molécule incriminée; Le biais de sélection: l'étude n'a concerné que les patients avec une HTA essentielle sans complication. Une autre limite a été le manque de coopération de certains patients. En effet, les rendez-vous manqués ou le refus de se faire interviewer pourraient avoir influencé les résultats obtenus. Le nombre de non répondants était de 82 patients. En outre, la non notification systématique des effets indésirables par le personnel soignant a pu limiter la détection des effets à partir du carnet de santé du patient. Enfin, l'imputabilité des effets indésirables à un médicament antihypertenseur a souvent été délicate du fait non seulement de la prescription des antihypertenseurs avec d'autres médicaments mais aussi de la similitude de certains symptômes de l'HTA avec ces effets indésirables. Afin de minimiser ces biais, nous avons d'une part, effectué un échantillonnage aléatoire et d'autre part, travaillé sur une population beaucoup plus importante pour améliorer la fiabilité de nos résultats.

### Caractéristiques sociodémographiques des patients

**Le sexe:** La population d'étude était caractérisée par une prépondérance féminine avec 69,1% de femmes, soit un sex ratio de 0,45 en faveur des femmes. Millogo et coll. en 2015 trouvait la même tendance (32,6% vs 27,1%) dans une étude sur le profil épidémiologique et le niveau de connaissance de la population adulte de 25 à 64 ans dans la ville de Ouagadougou [7]. Plusieurs raisons pourraient expliquer cette prédominance féminine dans les études hospitalières: les femmes consultent plus fréquemment dans les centres de santé (grossesse, contraception, dépistage des tumeurs du sein et du col de l'utérus); Les femmes sont plus sujettes à l'obésité [7] et à la sédentarité [12]; La prise d'œstroprogestatifs.

**Antécédents familiaux d'HTA:** Des antécédents familiaux d'HTA avaient été signalés chez 57,2% des patients de l'étude. Millogo et coll. [7], en 2015, rapportait un taux de 41%. Plus de la moitié des hypertendus avaient des antécédents familiaux d'HTA. Ces résultats sont en accord avec les données scientifiques qui évoquent un indice génétique dans la survenue de l'HTA; les enfants de parents hypertendus seraient plus à risque de développer une HTA.

### Caractéristiques des traitements suivis par les patients

**Nombre de molécules composant le traitement:** La répartition des patients enquêtés en fonction du nombre de molécules composant leur traitement donnait 43,2% sous monothérapie et 35,6% sous bithérapie pour ce qui concerne du traitement initial. La majorité des patients de cette étude était sous monothérapie et il s'agissait d'HTA non compliquée. Ceci est conforme aux recommandations de la Haute Autorité de Santé (HAS) [13,14] et de la société américaine d'hypertension qui suggèrent de toujours initier un traitement antihypertenseur par une monothérapie ou une association fixe d'antihypertenseurs à doses faibles. Selon la même source, la probabilité de contrôler un patient âgé sous monothérapie est d'environ 30%. Concernant la bithérapie, elle est recommandée lorsque la pression artérielle systolique est supérieure 180 mmHg ou lorsque la réponse tensionnelle est insuffisante au traitement initial après 4 semaines de traitement [13].

**Molécules et classes thérapeutiques utilisées pour le traitement:** La fréquence des molécules utilisées dans le traitement initial donnait les inhibiteurs calciques en tête (59,7%) avec la nifédipine (29,5%) et l'amlodipine (26,6%). Les diurétiques occupaient le second rang avec 43,9% et les IEC la troisième place (39,6%). Contrairement à nos résultats, chez Barbeau, [15] les diurétiques arrivaient au premier rang avec 83,4% des patients. Cette différence pourrait s'expliquer par le fait que son étude portait sur des personnes âgées et que les recommandations de prise en charge privilégient les diurétiques dans l'initiation du traitement chez le sujet âgé [13]. Cinq classes d'antihypertenseurs sont recommandées en première intention dans l'HTA essentielle. Il s'agit des diurétiques, des bêta-bloquants, des inhibiteurs calciques, des IEC et des ARA II. Le choix de la molécule sera fonction du niveau de risque cardiovasculaire et du terrain du patient [13]. Dans la pratique médicale au Burkina Faso, les initiations des traitements antihypertenseurs comportent très souvent de l'amlodipine ou de la nifédipine à faible dose compte tenu des contre-indications qui sont relativement peu fréquentes dans ce groupe.

### Prévalence et caractéristiques des effets indésirables

**Prévalence globale:** La prévalence globale des effets indésirables était de 60,1%, soit 167 patients ayant manifesté au moins un effet indésirable sur les 278 patients de l'étude. Barbeau [15] en 1998, retrouvait 48,9% de patients avec au moins un effet indésirable dans une étude portant sur des hypertendus sous monothérapie. La différence pourrait s'expliquer par le fait que son étude concernait exclusivement des patients sous monothérapie, donc susceptibles d'avoir moins d'effets indésirables. Au Nigeria, selon Olowofele et coll. [16] 18,1% des patients suivis dans un hôpital universitaire rapportaient au moins un effet indésirable lié au traitement antihypertenseur. Cette étude n'a pas pris en compte les effets indésirables observés par les soignants et reportés dans le carnet de suivi du patient. Ce qui peut expliquer une sous estimation de la prévalence. Les biais de mémoire pourraient affecter la prévalence établie sur la base des déclarations des patients.

**Répartition des effets indésirables selon les classes thérapeutiques incriminées:** Les inhibiteurs calciques étaient associés dans 53,6% des cas d'effet indésirable. Ils sont suivis des diurétiques (48,6%) et des inhibiteurs de l'enzyme de conversion (IEC) avec 48,2%. L'étude de Olowofele et coll. [**16**] classe les diurétiques au premier rang des médicaments les plus fréquemment impliqués dans les effets indésirables suivis des inhibiteurs calciques. L'amlodipine (28,1%), l'hydrochlorothiazide (24,5%) et le captopril (22,3%) étaient les molécules qui avaient le plus occasionné l'apparition d'effets indésirables. Ces trois molécules correspondent aux trois classes thérapeutiques les plus utilisées dans la prise en charge de l'hypertension artérielle dans cette file active de patients.

**Répartition des effets indésirables selon la nature de l'effet indésirable:** La diurèse excessive (13,7% des patients), la toux sèche (12,9%) et les vertiges (11,5%) ont constitué respectivement les types d'effets indésirables les plus observés. Olowofele et coll. [16] avaient rapporté les mêmes effets indésirables en plus de céphalées. Tous ces effets sont en relation avec les classes thérapeutiques les plus prescrits: inhibiteurs calciques, diurétiques, IEC. Il s'agit donc d'effets indésirables attendus avec certaines classes thérapeutiques. Mais leur fréquence nécessite plus d'attention de la part des prescripteurs pour améliorer le confort du patient durant le traitement. En termes de proportion de patients ayant manifesté les effets indésirables par appareils ou systèmes, cela représente respectivement 19,1% pour le système nerveux central et périphérique et le système ostéo-musculaire et 16,9% pour l'appareil respiratoire.

## Conclusion

Cette étude montre bien la réalité et l'importance des effets indésirables des traitements antihypertenseurs chez les patients suivis en ambulatoire. Les effets indésirables sont un déterminant majeur de l'adhésion aux traitements. Aussi, si la plupart des effets indésirables semblent modérés ou banal (diurèse excessive, vertiges, toux), leur impact sur la vie quotidienne des patients pourrait s'avérer significatif. Il serait intéressant de développer l'éducation thérapeutique auprès de cette catégorie de patients chroniques afin d'améliorer la détection et la gestion des effets indésirables.

### Etat des connaissances actuelle sur le sujet

- Les traitements médicamenteux entrainent de nombreux effets indésirables qui rendent souvent l'adhésion au traitement très difficile;
- Les effets indésirables rares et potentiellement graves sont peu observés, après les essais cliniques, pour la mise en circulation des médicaments antihypertenseurs.

### Contribution de notre étude à la connaissance

- Une bonne connaissance des effets indésirables du traitement antihypertenseur afin de permettre d'une part, de faire le meilleur choix dans l'option thérapeutique et d'autre part, d'éduquer le patient sur la survenue de ces effets indésirables ainsi que sur leur gestion afin d'améliorer l'adhésion et l'observance du traitement par le patient.

## Conflits d’intérêts

Les auteurs ne déclarent aucun conflit d'intérêts.
